# Designing pain visualisation for caregivers of people with special needs: A co-creation approach

**DOI:** 10.1016/j.heliyon.2022.e11975

**Published:** 2022-12-05

**Authors:** H. Korving, P.S. Sterkenburg, E.I. Barakova, L.M.G. Feijs

**Affiliations:** a Department of Behavioral and Movement Sciences, Clinical Child and Family Studies, Amsterdam Public Health, Vrije Universiteit Amsterdam, Van der Boechorststraat 7, 1081 BT, Amsterdam, the Netherlands; b Bartiméus, Oude Arnhemse Bovenweg 3, 3941 XM Doorn, the Netherlands; c Department of Industrial Design, Future Everyday Group, Eindhoven University of Technology, Groene Loper 3, 5612 AE Eindhoven, the Netherlands

**Keywords:** Daily caregivers, Interface design, User-centred design, Collaborative design, Perception

## Abstract

Recognizing pain in people with communicative disabilities is challenging. A support system detecting pain signals provides caregivers with information to intervene adequately. This study aims to develop a design for a user interface visualizing pain experiences for a signalling system intended for caregivers. Caregivers receive alerts, indicating the presence or absence of pain experienced by a disabled individual. The design process included the use of value proposition, a brainstorm, a mood board with basic design elements, and multiple questionnaires and focus groups. During the multi-disciplinary design process end-users were extensively involved. The final design was deemed intuitive, clear and recognizable, and useable in daily caregiving. This article describes the creation process for a non-hedonistic visualization for this niche end-user group.

## Highlights

• In this article a complex, hard to recognize and challenging concept (pain) is visualized.
• A multi-disciplinary co-creation approach was used, involving end-users in each aspect.
• The end-users consist of a niche user group on which very few studies have focused.
• The resulting design is intuitive to the end-user and can be implemented in a real-life setting.

## Graphical abstract

**Figure:**
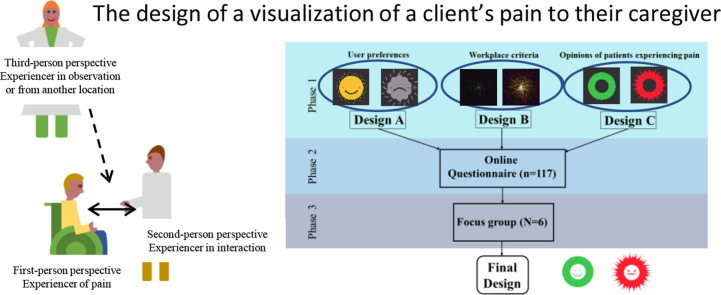

## 1 Introduction

When a person is unable to express pain, as is the case for those with severe or profound intellectual disabilities (S/PID), this poses a challenge for caregivers (Griffiths and Smith, 2016). Especially when a genetic disorder also brings about a multitude of painful medical afflictions, which is often the case (e.g. Doody and Bailey, 2006; Walsh, Morrison & McGuire, 2011), the necessity to spot the pain timely and adequately increases. Due to S/PID, clients often display subtle or idiosyncratic signs of distress (e.g. freezing, laughing) and they depend on caregivers for pain and stress relief (Van der Putten and Vlaskamp, 2011). Some of the most frequent painful disorders, such as gastro-oesophageal reflux and obstipation, are easily treatable, which makes inaccurate or absent pain recognition all the more poignant. Caregivers could use aids to help them become aware of pain among those unable to clearly express it.

To provide caregivers with information about the existence of pain in their clients with S/PID, a system was developed that uses physiological signals to measure possible pain (Korving et al., 2020). The system utilises a sock with sensors for skin conductance, a transmitter sending and receiving information from the sock sensors and sending it via Bluetooth™ to a mobile application on phone or tablet (Frederiks et al., 2015; Frederiks et al., 2019). The mobile application contains a AI-enabled algorithm that was trained to distinguish physiological pain signals from other signals (Korving et al., 2022). This article focuses on the development of the visualization accompanying the warning in the mobile application.

### 1.1 Related work

**Figure 1:**
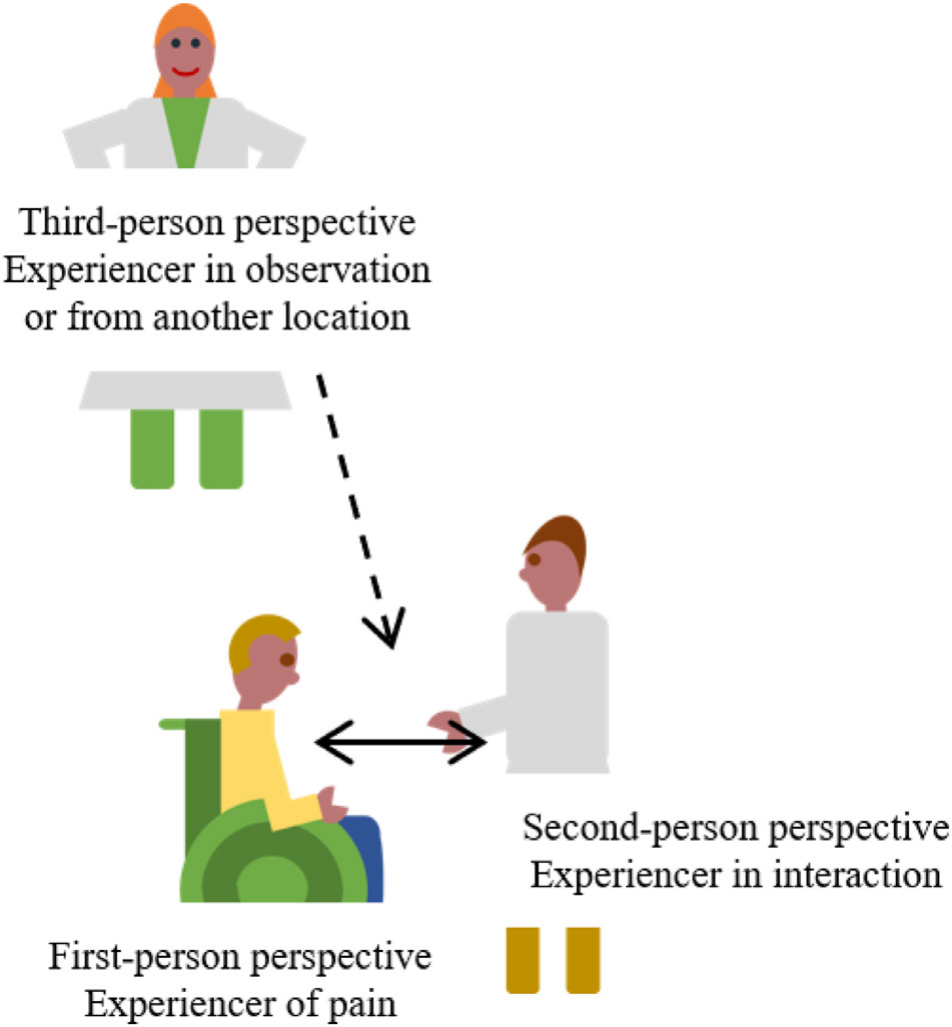
A graphical representation of the three perspectives correlated in the visualization developed this study.

The use of technology in healthcare is growing and data is routinely collected, which becomes valuable for use in the design for new care solutions (e.g. Bughin, 2016; Chen & Floridi, 2013). Transforming the available data to understandable, timely, and meaningful information for users is of crucial importance. The transformation of data is especially challenging when there are multiple users with different perspectives and a multitude of experiences which shape the appraisal and managing of other's pain (Craig, 2009, p. 24). In terms of phenomenology, the philosophical study of experience and consciousness (Price et al., 2002; Thacker and Moseley, 2012; Zahavi, 2003), the first-person perspective of pain is the person who is experiencing pain, in this case, an individual with S/PID, who is unable to clearly express this. Those who interact with the individual in pain have a second-person perspective on the pain experience. This will be the caregivers of the individual with S/PID. Those that do not interact with the client, but observe an interaction, for example researchers and other care professionals, have a third-person perspective on the experience of pain. The application to be developed translates the first-person experience and displays that to someone with a second- or third-person perspective (Figure 1).

To signal this first-person pain perspective to another a visualization can be used. The use of technology in aid only emanated in the past half-century, but information has been visualized since there was a need to share it (Birx, 2006). The underlying data might change, but the need to visualize information remains. Considering a limited amount of data can be stored in working memory at one time (Van den Berg et al., 2014), when more data are presented receivers get confused, make errors and forget items (Maehara and Saito, 2007). When compacted data is shown in drawings or charts, not only does information become clearer, drawings are “[…] more effortless to recognize and process than words, but also easier to recall” (Dewan, 2015, p. 2). In a busy and distracting caregiving environment, caregivers have limited time to perform a variety of tasks for a variety of clients and simultaneously attend to many aspects of their well-being: A simple and intuitive aid to help with one of these aspects will be invaluable.

Visualization of information is also preferred when it provides ambiguous or pre-analysed information. An example of this is data on physical processes. Especially when the goal of physical measurement is information on health status or the presence of an illness, more information can enhance interpretation, but more data may reduce clarity (Bughin, 2016). A dynamic and simple visualization gives sufficient information to a health care provider, without requiring too much cognition or providing too much information for understanding.

### 1.2 Gaps in knowledge

An experience of pain is difficult to capture with standardized numeric measurement tools, such as the Numeric Pain Rating Scale (Jensen et al., 1986; Gooberman-Hill et al., 2007; Wylde et al., 2012). Thus, over time, several colour- and shape-based tools were developed and used for assessment of pain in children, pain from burn wounds and chronic pain (e.g. Grossi et al., 1983; Machata et al., 2009; McGrath et al., 1996). These non-numeric pain assessment tools were validated and compared to numeric scales and results indicated that patients preferred the former to describe a pain experience over the latter. However, studies validating non-numeric based assessment tools used pre-determined colours and shapes/figures without examining whether these accurately represent the pain experience for users. Therefore, it is unknown if visualizations used in these scales are intuitive for the pain experience. The current study bridges this gap by describing a process to ascertain the intuitive colours, figures, shapes and characteristics associated with pain in the user group.

In the Netherlands, a caregiver usually cares for more than one person with S/PID, and a person with S/PID is cared for by more than one caregiver. Since caregivers want to provide high-quality care and attend to the needs and challenges of persons with S/PID, interpretation of the information in the app should be accurate and easy to understand. Furthermore, since multiple caregivers care for one disabled individual, inter-pretation of the data should be user-independent (Jansen et al., 2013; Kruithof et al., 2020). Above all, a caregiver's workplace provides challenges to incorporate a system, therefore one design challenge addressed in this article is converting numerical data to a visualization that is recognizable by many different users with different levels of cognition which corresponds to pain experienced by adult clients with S/PID, which is practical to use within a distracting working environment.

### 1.3 Study objectives

The aim of the current study was to develop a visual design of pain, which changes in colour, shape and/or characteristic to indicate whether pain is, or is not, present. The design was developed in co-creation with persons from second- and third-person perspectives, meaning that the visualization is co-developed with caregivers who experience the pain of the person with S/PID by interaction as by care professionals and other persons who observe the interaction. This study addresses the following questions: 1. What are visual associations with pain with respect to shapes, colours, figures and characteristics? 2. What are factors to be considered when designing visualizations to be used in the workplace? 3. What design for pain can be made based on a literature search of visual associations of pain from patients with (chronic) pain? 4. What are preferred characteristics of visualizations with respect to clarity, distinguishability, intuitiveness and pain association?

## 2 Design process

The goal of the design co-creation process was to create a concept for a user interface that visualizes pain applicable in daily caregiving. Accuracy and intuitiveness of the visualization was tested, as well as its usefulness in caregiving situations. Caregivers of persons with S/PIMD, the intended end-users of the app in which the visualization is shown, were included in every aspect of the research, and collaborated throughout the design process up to and including the creation of the final concept.

**Figure 2:**
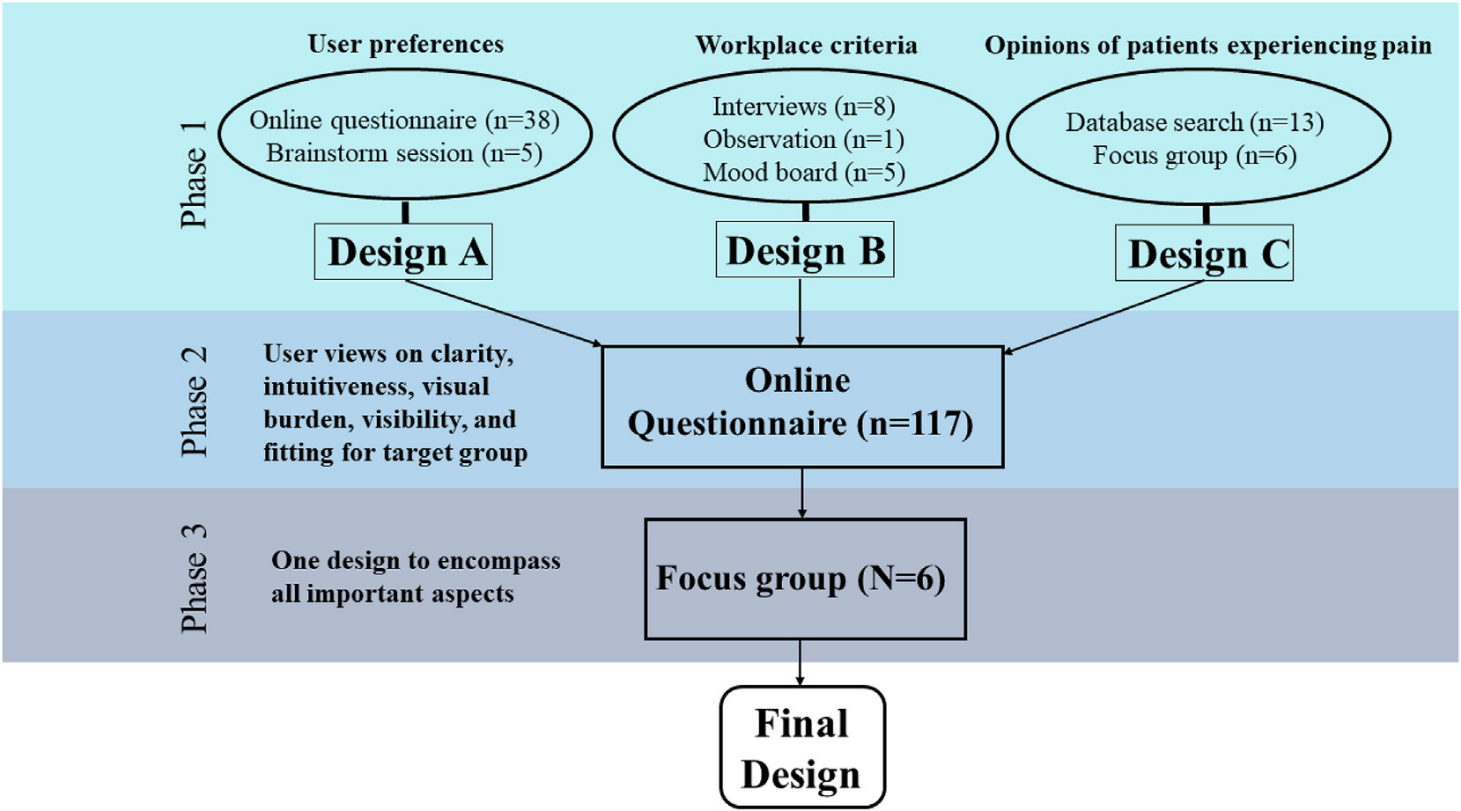
A visual display of the design process; showing the sub-steps leading to three initial designs and the three phases of the development of one final design.

The process consisted of three phases, displayed in Figure 2. Phase one included three experimental approaches, performed in parallel, each containing at least two rounds of consultations, in order to create three different designs. The reason for this choice was to keep the involvement of individual caregivers as brief as possible and keep the design aspects to which they had to contribute as simple as possible. Design A was based on end-user preferences regarding basic form aspects, which address their associations to pain. Design B was based on workplace criteria, which address the challenge of designing for tasks in daily caregiving. Design C was based on peer-reviewed literature on qualitative data from (chronic) pain patients, which address the need for simplicity and intuitiveness. The involvement of end-users in every step of the design process has been immensely valuable and has aided into making the design process intuitive and attuned to the end-users’ needs.

In phase two, an online questionnaire to evaluate the three designs created in phase one was administered among the user group. Users evaluated each of the three designs on a variety of usability aspects, such as clarity, distinguishability, intuitiveness and pain association. The challenges of phase one were addressed once more with the addition that designs were mutually compared regarding usability. In phase three an expert focus group was presented with the results of the questionnaire from phase two and a final design was selected which encompassed all aspects from phases one and two. This elaborate design process was used on account of the lack of user research in this very specific target group and the unique needs and challenges professional caregivers for clients with S/PIMD face in daily caregiving (Van Timmeren et al., 2017a, 2017b).

The design study described in this article is part of a larger study to develop a system to physiologically measure pain in adults with severe or profound intellectual disability. The overall study was approved by the medical ethics board of the Vrije Universiteit Medical Centre in 2019 under no. NL69815.029.19.

### 2.1 Participants

In this study different experts were asked to contribute their opinion, expertise and ideas on a possible pain design. Most of these experts will eventually also serve as end-users for the system which will contain the visualization. Experts that will possibly not be end-users were the design students and professor, who participated in the brainstorm session of phase 1.1.2 and the researchers with expertise in research among people with severe and profound intellectual disability (SID). Other experts ranged from parents of children and adults with SID to speech therapists and medical personal working with people with SID. Table 1 gives an overview of the experts and their respective fields of expertise per phase of the process.

**Table 1:** Description of expertise per expertise group for each part of the design process.

Phase	Subphase	Part	Expertise	#
1	1.1	1 1.1.1 (N = 38)	Parent of SID	8
			Daily care for SID	30
		1.1.2 brainstorm (N = 5)	Design	4 students, 1 prof
		1.1.2 value points	Research in SID	2
	1.2	Design criteria (N = 9)	Daily care for SID	5
			Research in SID	3
			Medical personnel	1
		Mood board (N = 4)	Daily care for SID	2
			Research in SID	2
		Evaluation	Daily care for SID	3
	1.3	Review	Research in SID	1
		Focus group (N = 6)	Daily care for SID	3
			Research in SID	2
			Medical personnel	1
2		Questionnaire (N = 117)	Parent of SID	15
			Daily care for SID	33
			Behavioural therapist	24
			Physical therapist	24
			Speech therapist	6
			Medical personnel	6
			Other experts in care	9
3		Focus group (N = 6)	Daily care for SID	3
			Research in SID	2
			Medical personnel	1

Due to COVID-19 restrictions, most of the recruitment and execution of the study was done online. For recruitment, the project sent out newsletters to interested parties with an appeal to fill out online questionnaires. Participants for focus groups and design criteria parts were recruited through the network of first and second author; two national organizations for the care of people with (visual and) intellectual disability collaborate as partners. The design students were recruited through the network of first and last author at Eindhoven University of Technology.

### 2.2 Phase 1.1: from user preferences to design A

Design A was based on user preferences regarding the relation of shapes, colours, figures, and graphical features to an experience of pain. These associations were selected by end-users using visuals in an online questionnaire. The results from the questionnaire were then presented to a group of Industrial Design students in a brainstorm-design-session. Designs made in the brainstorm session were then assessed on aspects from the Value Proposition Tower from Road2Results (Road2Results, 2020, Figure 4). Based on their value scores consensus was reached on the best-scoring designs and Design A was created.

Subphase 1.1.1 Online questionnaire to assess user preferences (design A).

#### 2.2.1 Methods

A comprehensive online questionnaire was distributed among those providing care for persons with S/PIMD in the Netherlands, using Qualtrics survey software (Version XM of Qualtrics. Copyright © 2020 Qualtrics, Provo, UT, USA). In the questionnaire, participants compared several items on their association with the experience of pain or ‘no pain’. Again, this design process, which might seem superfluous, was adopted to correct the overall lack of user research in this niche target group.

**Figure 3:**
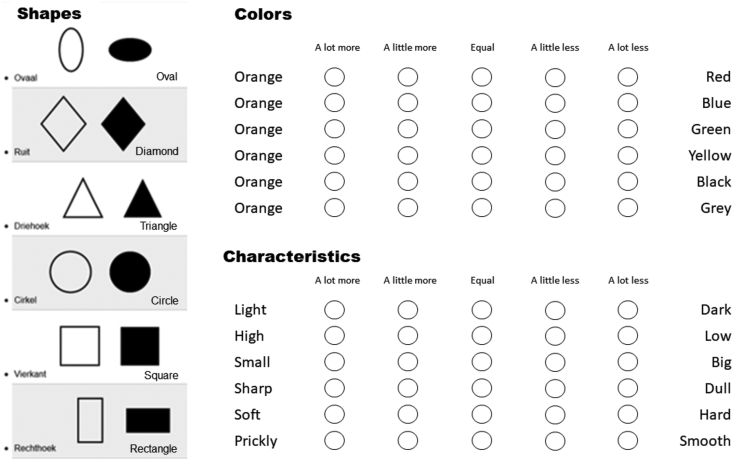
An example of two different assessment methods presented in the online questionnaire of basic aspects. The six provided shapes on the left were ordered from highest association with pain (top) to lowest (bottom) and the colours and characteristics were compared to counterparts on a five-point scale.

The questionnaire items were divided among four categories: “shapes”, “colours”, “figures” and “characteristics”. End-users were asked to either order different items from least to most associated with pain or to individually compare one item to another of a similar category. Figure 3 shows an ordering system for six different shapes and a comparison system for the colour orange and six characteristics. All categories and the items within were presented in random order.

**Figure 4:**
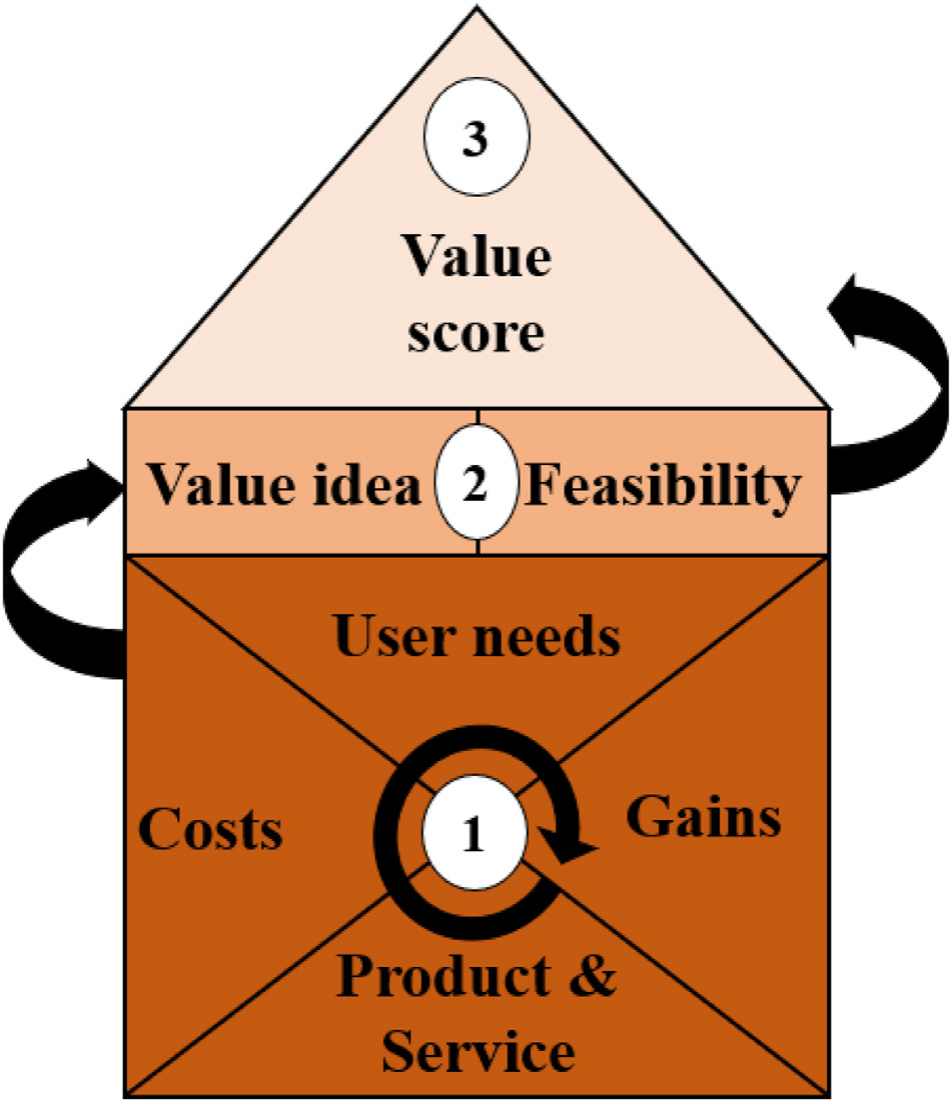
This Value Proposition Tower shows a process by which each idea is assessed on two levels, starting at the bottom (1) with a iterative route through design possibilities (Products & Service, Costs, User Needs and Gains), which are combined in each idea's initial ‘value’ (2), which, combined with its feasibility, results in an eventual value score (3).

In the categories “shapes” and “figures”, participants were asked to order six shapes and seven figures from the highest association to pain at the top to the lowest association at the bottom. In the “colour” category, every colour was compared to all other colours in the questionnaire, via a semantic differential method (Osgood et al., 1957). The participant decided whether a colour was ‘a lot more’, ‘a little more’, ‘equally’, ‘a little less’ or ‘a lot less’ associated with pain compared to the other colour (for an example, see Figure 3). The category “characteristics” consisted of fifteen comparisons between pairs of polar opposites, such as ‘light’ and ‘dark’ and ‘smooth’ and ‘prickly’. These were shown in a grid horizontally opposite each other. Participants scored these pairs on the same 5-point scale as colours (Figure 3). For each item, a z-score was then calculated, using Microsoft Excel, based on averages and standard deviations of all items in that category. High positive z-scores signify a higher association to pain by participants compared to items with lower positive or negative z-scores. Negative z-scores signify an association with ‘no pain’.

#### 2.2.2 Results

For each category in the questionnaire, both the highest scoring two items and the lowest scoring two items are presented in Table 2. Results from z-score calculations indicate that the triangle shape was most associated with pain, while rounded shapes were not. This result is mirrored in the characteristics category. Red was most associated with pain and green with ‘no pain’. From the list of seven possible figures, the ‘sad face’ was chosen as most associated with pain. Participants that gave their own suggestions of shapes associated with pain mentioned the spiked shape/star (7 participants, 23%) and the lightning bolt (6 participants, 20%).

**Table 2:** Items in the questionnaire that had the highest and lowest associations with pain from z-score calculations for 30 participants.

Shapes				Colors			
High		Low		High		Low	
Triangle	2.49	Circle	0.62	Red	1.42	Green	-0.91
Diamond	2.37	Oval	0.62	Orange	0.44	Blue	-0.58
Figure				Characteristics			
Sad face	2.44	Heart	0.32	Prickly	1.14	Smooth	-1.14
cross	2.19	Flower	0.48	Rought/Hard	1.09	Fluffy/Soft	-1.09

NB. The categories shapes and figures were only given positive scores, so the score zero represents more association with ‘no pain’, while the categories colours and characteristics were given positive or negative scores, so negative higher scores represent more association with ‘no pain’.

Subphase 1.1.2. Brainstorm session and value calculation based on questionnaire results.

#### 2.2.3 Methods

To interpret and expand on results from the online questionnaire, a creative brainstorm session was held with students and a professor from a bachelor's program in Industrial Design. During the 60-minute session the ‘designing for extreme scenarios’ and ‘designing for extreme characters’ (Djajaniningrat et al., 2000) methods were followed, led by an experienced session leader. These ‘extreme’ forms use fictional situations and end-users in an excessive manner to let designers consider, among other things, the social role of their designs and how end-users will physically interact with the eventual product.

The design experts were asked to assess the results from the questionnaire in terms of their design ideas; keeping in mind three design requirements:

• The visualization should clearly distinguish between a ‘pain’ and a ‘no pain’ level;
• The design should be visually clear for those with vision impairments and mono-chromatic vision; and
• The design should demonstrate when data is being received (e.g. by moving).

Participants in the brainstorm were first asked to freely associate pain-related concepts. They were then asked to design a visualization for a man of around 80 years old, as an example of an extreme character. Next, they were asked to design regarding possibilities that may be possible ten years in the future, as an extreme scenario. Finally, participants presented their designs and were asked to build upon each other's ideas (Design Kit, Ideo.org, 2020). A discussion was initiated about the usefulness of all design ideas generated in the brainstorm in practice now and in the future. First author attended the brainstorm, and collected and categorized all ideas.

First and second author evaluated the design ideas from the brainstorm one by one using a value proposition tower from Road2Results (Road2Results, 2020). This evaluation process, shown in Figure 4, aided in developing a viable value proposition in a structured way. Each design idea presented in the brainstorm session was initially evaluated on four basic criteria: Product & Service, Costs, User Needs, and Gains. Product and Service entailed what can realistically be offered by a designer in terms of services. It established the function and constraints of the product. The User Needs were constraints of the design possibilities based on needs of the end-users, for example constraints of a caregiver's working environment (noisiness) or their attention span. Costs were aspects that make an idea less viable to be used or appreciated by end-users, and Gains were idea-aspects that increased its value as a concept. Costs and Gains were assessed based on the outcome of Product & Service and User Needs in an iterative process.

An example of a design's cost is a general negative association with the visual, something that may be perceived as insensitive due to personal beliefs, or something that is open to interpretation. Examples of gains are multiple modality changes for better visibility, clear difference between the negative and the positive poles and recognizability. Of course, a design can both have costs and gains, meaning it can be recognizable as well as culturally insensitive. The final value of each design idea (Value at the top of Figure 4) was based on the combined result of the four criteria mentioned before (Value idea at step 2 in Figure 4), augmented by how feasible the idea was to become a product (Feasibility).

#### 2.2.4 Results

Each participant in the brainstorm session created three to five designs, some of which were interchangeable or unfeasible, which resulted in thirteen ideas, presented in Table 3 as simplified descriptions. Scores in the table are based on the value proposition assessment described above. Each cost received a score of -1, when present, or 0, when absent and each gain received either a 1 or 0. An idea's Value score is the total score, evaluated on five possible costs, from -5 to 0, and seven possible gains, 0 to 7.

**Table 3:** List of ideas generated in the brainstorm and their results from the value proposition.

Idea	Content	Costs∗	Gains∗∗	Value score (sum)
#1	Happy face - > sad face	0	6	6
#2	Whole body in pain	-2	6	4
#3	Graphical display	-2	4	2
#4	Small dot - > large circle	-1	3	2
#5	Religious characters	-4	2	-2
#6	Military aspects	-4	2	-2
#7	Shapeless blob - > spikes	-1	3	2
#8	Good - > bad weather	0	6	6
#9	Smooth oval - > spiky diamond	0	3	3
#10	Moving line	-2	4	2
#11	Spiral - > spikey graph	-1	3	2
#12	Bubble - > lightning sparks	0	3	3
#13	Low - > high saturation	-3	0	-3

∗ such as negative associations, culturally impractical, similar, open to interpretation, etcetera.

∗∗ such as clear, intuitive, easy to recognize, distinguishable, usable in colour blindness, etcetera.

**Figure 5:**
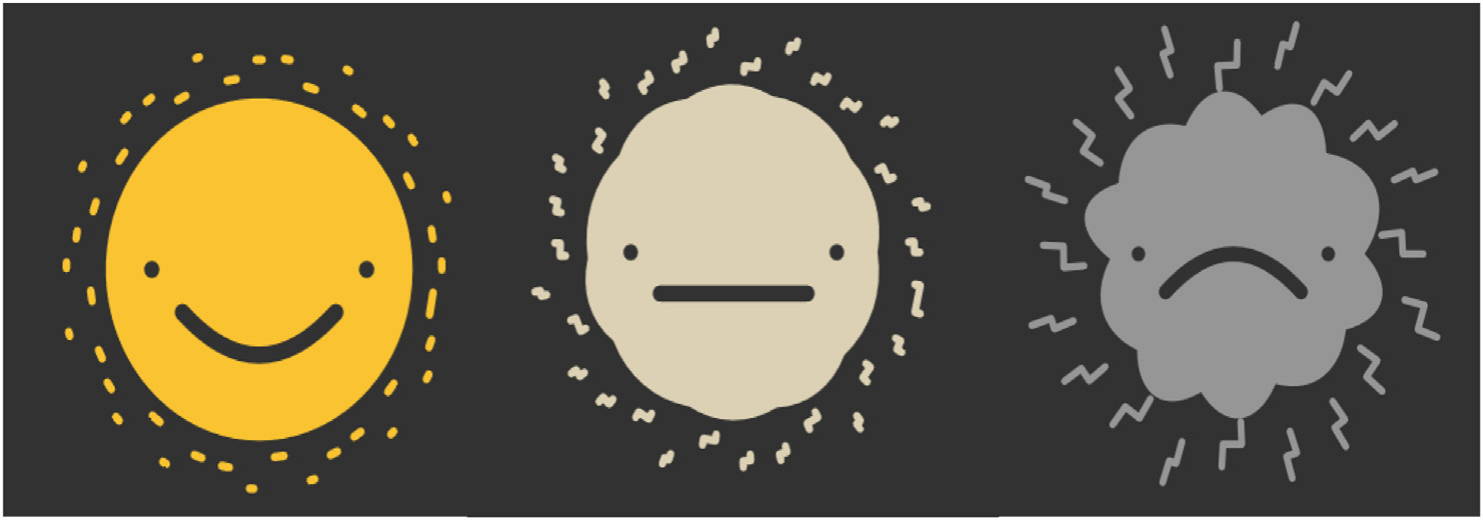
Design A: the result of subphase 1.1. A yellow and smiling sun represents ‘no pain’ and a sad storm cloud represents ‘pain. The visual in the middle shows in what way the visualization transfigures from a sun to a storm cloud.

With the result from the value proposition, two ideas came out as best scoring: ‘the happy and sad face’ (#1) and ‘good to bad weather’ (#8). Authors decided to combine the two ideas in one design, with a shape that transforms from a round sun to a storm cloud and a colour change based on weather conditions: Yellow for good weather/sunshine and grey for bad weather/storm and lightning. The face alters from smiling to sad, as shown in Figure 5.

### 2.3 Phase 1.2. from workplace criteria provided by caregivers to design B

#### 2.3.1 Methods

**Figure 6:**
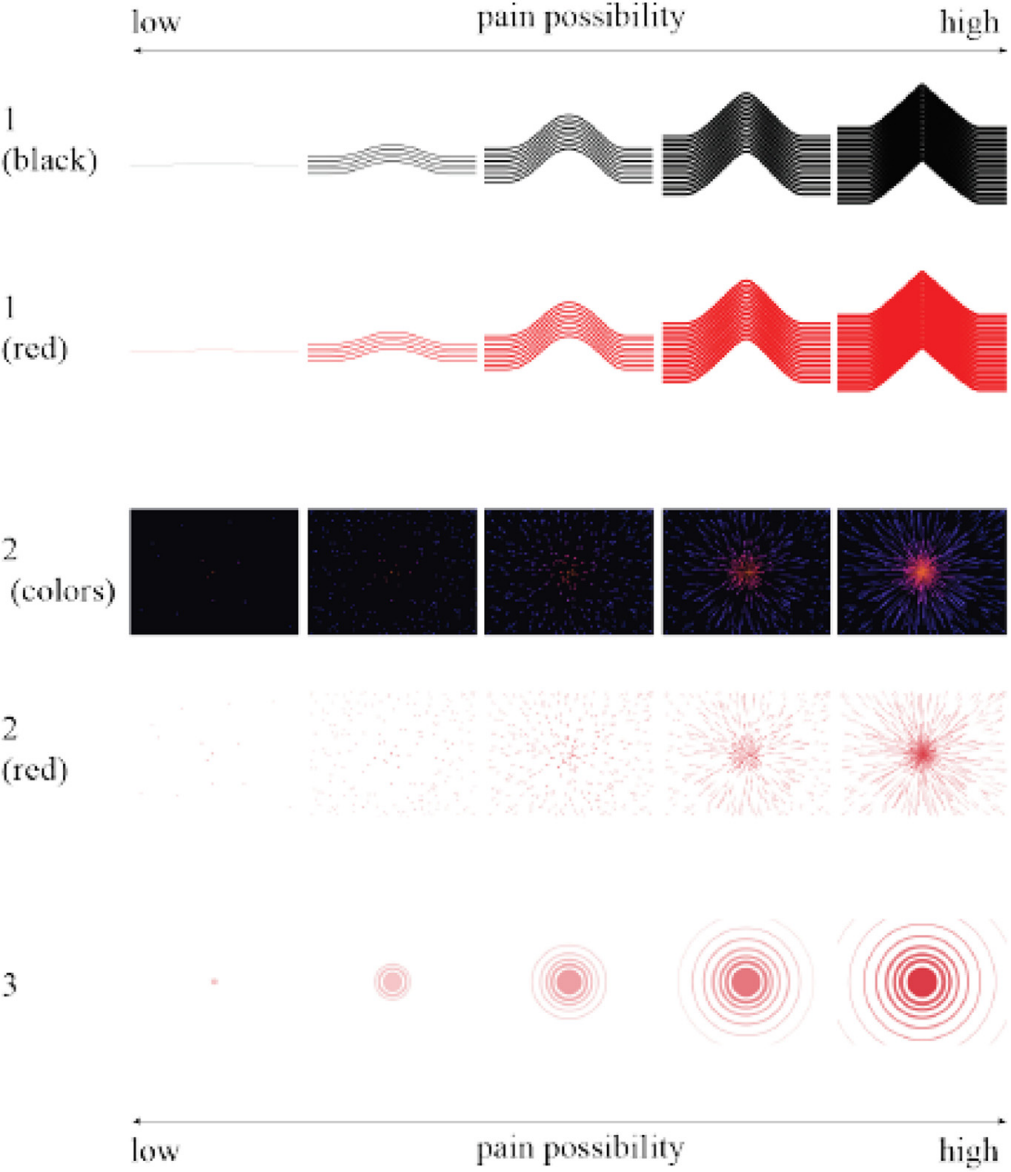
The mood board created for evaluation in the process to create design B displays three basic design ideas, which were assessed by caregivers, therapists and researchers (Zhang et al., 2021).

**Figure 7:**
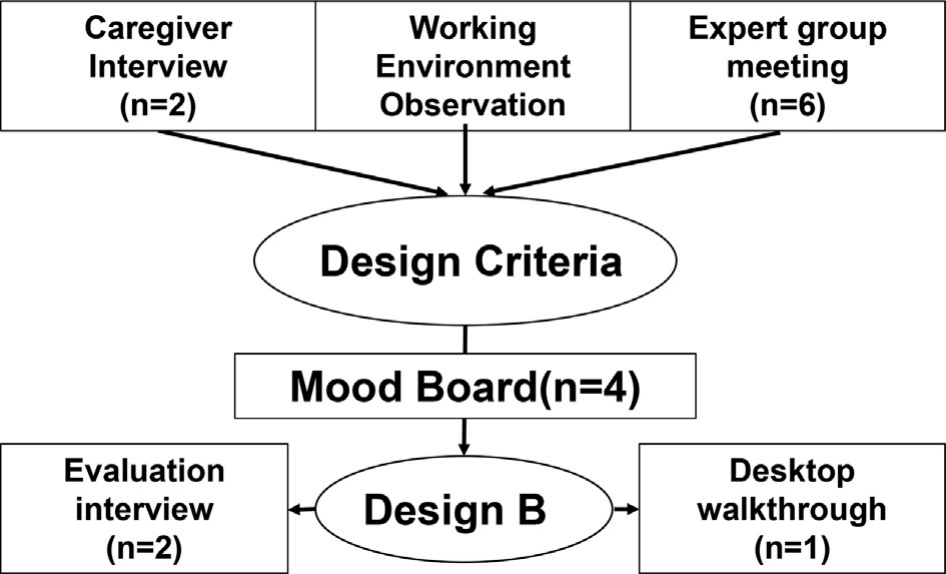
The process for design B includes two caregiver interviews, an observation in the workplace and an expert group meeting, followed by a mood board with basic design criteria. The final sub-design is evaluated in two interviews and a workplace walkthrough (Zhang et al., 2021).

Design B was created in collaboration with caregivers in their daily workplace environment. Caregivers from different care organizations were either interviewed or observed at work by a research assistant to assess work-related needs for the visualization. Afterwards, an expert group meeting was organized to translate these needs into design criteria. Based on these first steps, a mood board with nine pain-related basic design images was created to study experts’ mental models on perceiving pain from visual elements. The mood board was presented to caregivers, therapists and pain researchers, who were asked to rate the design images on two axes: ‘comfortable-painful’ and ‘attention drawing-easy to ignore’. In addition, experts assessed three basic designs, shown in Figure 6 (lines in red or black, spreading dots in red or various colours and growing circles in red), on provisions necessary to integrate the system into caregivers' workplace. Design B was created based on all the preceding steps. To assess its usefulness, the design was then evaluated in two interviews and assessed through a workplace walkthrough. The design sub-process is displayed in Figure 7.

#### 2.3.2 Results

**Figure 8:**
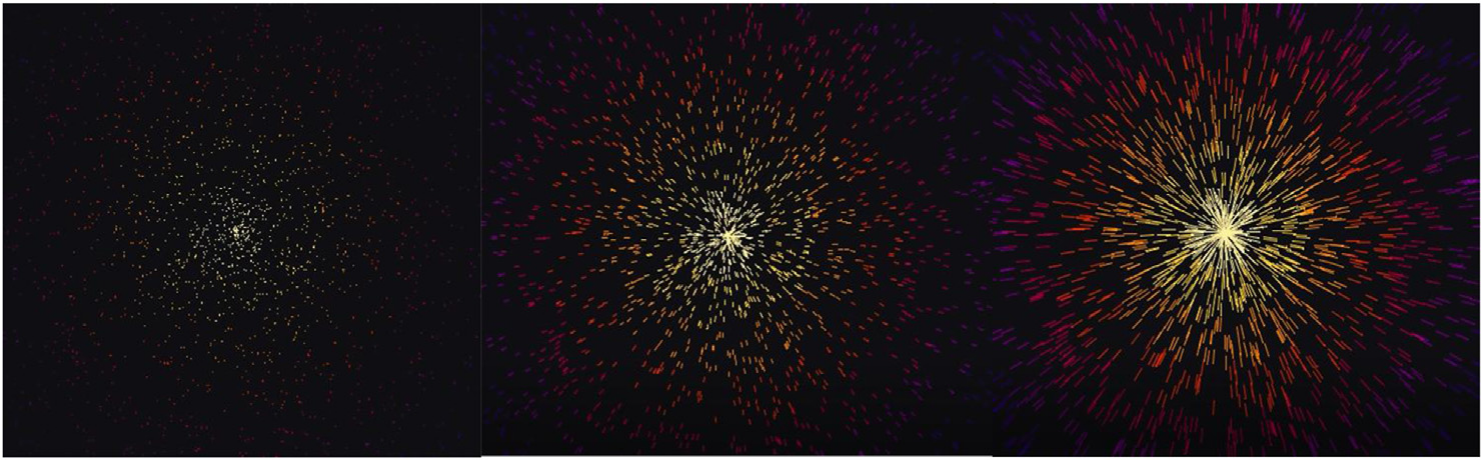
Design B is the result from subphase 1.2. From a focal point in the middle of the design, multi-coloured dots spread out into lines when the pain-level rises.

Experts who evaluated basic designs had similar opinions on the association with pain. They believed the first concept (number 1 in Figure 6) was ‘hardly’ associated with pain. Half the experts could not decide when to take action. The second concept (number 2 in Figure 6) was most associated with pain, though difficult to understand and found to have the highest mental burden. Experts found the third concept (number 3 in Figure 6) simple and easy to understand. It reminded them of alertness, not pain. This design would succeed to attract caregivers' attention most. Half the experts had concerns with the use of the colour red, and one believed it would help understanding. The multi-colour scheme of the second concept received the most positive feedback. This second concept in the multi-colour scheme was then chosen as design B, in a simplified and improved iteration, resulting in Figure 8.

The evaluation of this design by two caregivers showed that the design had an association with pain but would not create (extra) stress in a caregiver. After a short explanation, caregivers found the design easy to use. Awareness of the design was mixed, with one caregiver being very sensitive to changes in the display, while the other was less sensitive. Both evaluated the design in its ‘no pain’ state as not distracting them from computer-related tasks.

### 2.4 Phase 1.3. from opinions of patients experiencing pain to design C

#### 2.4.1 Methods

The third design was created based on results of peer-reviewed literature following a search on colours and visuals associated with pain in different databases (i.e. Worldcat.org, PsycINFO, Wiley Online Library, ScienceDirect, & ABI/INFORM). Search terms were “pain or nociception or burden or distress” and “colour or colour or visual or vision” in title and keywords. The words “shape” and “figure” were omitted on purpose, for these are commonly used as verbs in titles. The results of the search were ordered and checked on eligibility for the study. Original articles and reviews were included if they: (1) Focused on pain experiences of pain patients, (2) Discussed visual associations of pain, and (3) Results were systematically analysed.

Articles were ordered according to the type of visualization and then on methodology and participant size. Results were categorized in a table presenting the amount of consensus of participants, free associations (if asked), and limitations and implications of the results mentioned by authors. With the result of the literature search, first author chose basic design aspects that were most commonly found in literature and had few limitations, with the aim of creating a third design to be assessed in phase two.

The design based on literature was presented to a focus group of experts in the care for persons with intellectual disabilities. This expert group consisted of six persons: one general practitioner specialized in the care for persons with an intellectual disability, a professional caregiver, a scientist practitioner, a behavioural therapist and coordinator of a national platform for research on persons with severe intellectual disability, a researcher on arousal in persons with an intellectual disability and a behavioural therapist and professor on research in persons with visual or visual and intellectual disabilities. The group members were asked to relay their personal experience in their working environment and discuss possible implications of using the design as a visual display of pain in daily practice.

#### 2.4.2 Results

The literature search generated 2,133 results, of which titles and abstracts were reviewed based on inclusion criteria, resulting in 11 useful articles. Reference lists of relevant articles were scanned, after which a discussion with a design expert took place, leading to the inclusion of two additional articles. Of these 13 articles, ten (77%) evaluated pain associations based on colour, or colour and another aspect. The other three evaluated free pain associations: participants were asked to mention any aspect they could conceive.

Red was most often associated with pain (e.g. Altan et al., 2019), while Wylde et al. (2013) found black/grey associated with persistent pain and red with intense pain. Colours associated with no pain varied from light pink (Grossi et al., 1983) to yellow (Machata et al., 2009) and cool colours such as green (Palmer and Schloss, 2010), although these were also associated with illness (Elliot, 2015). Light and saturated colours were more positive than dark and muted colours (Palmer and Schloss, 2010). Other aspects used to indicate pain were wideness (wider equals more pain; McGrath et al., 1996), size (bigger equals more pain; Jackson et al., 2007) and sharpness (sharper equals more pain; Lootens and Rapoff, 2011). No pain was represented by a happy or neutral face and pain by a sad or crying face (Machata et al., 2009; McGrath et al., 1996). In the study by Lalloo et al. (2014) forms of pain were associated with sharp edged items, such as needles. The study by Lor et al. (2019) resulted in objects and animals as metaphors associated with pain.

**Figure 9:**
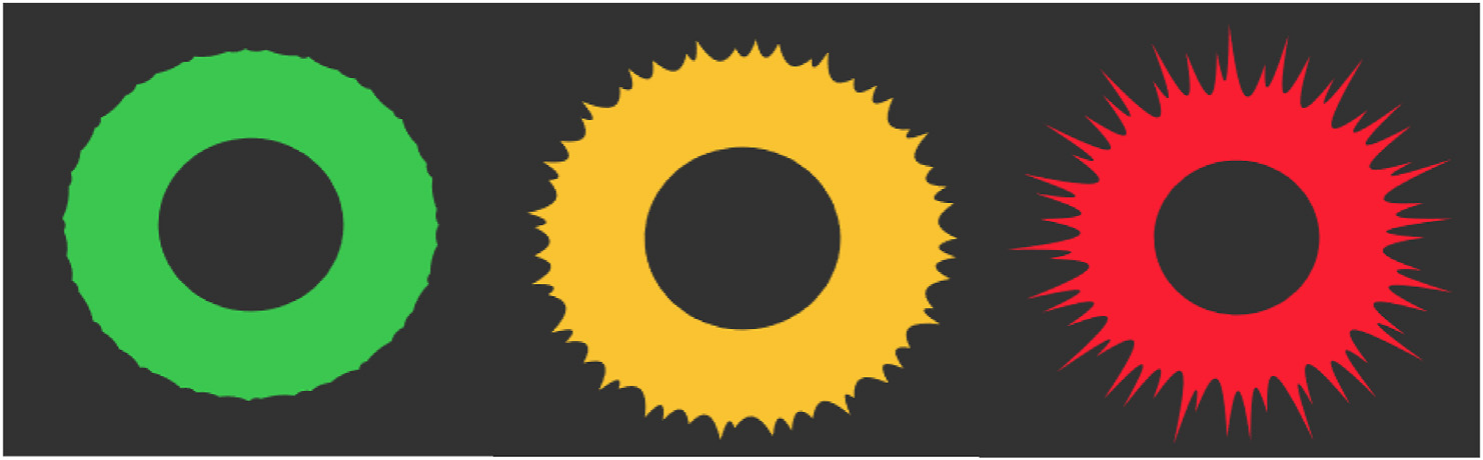
Design C is the result from subphase 1.3. The visualization shows a green circle with a rounded edge, slowly waving, which represents ‘no pain’, whereas a red circle with sharp spikes represents pain. The colours are adapted to those of a traffic light.

First author chose to use red for pain and green for no pain, partly to create a stop-and-go association in caregiving: A caregiver's action can continue as is at green, while red means the caregiver should stop and assess the client's well-being. Since sharpness was also intuitive for pain, this character was added. The design based on these results is displayed in Figure 9. An expert group evaluated this design as intuitive and understandable. The experts were content with its limited complexity, one visual for pain and one visual for no pain, which would make responses from caregivers upon seeing the visualization most reliable.

### 2.5 Phase two: comparison of the three designs on several important aspects

#### 2.5.1 Methods

In phase two, the three designs (A, B and C) were compared by a group of end-users. The visuals were played as moving gif-images varying from ‘no pain’ to pain, by changing shape, colour and/or property. The designs were distributed in a network of parents and professional caregivers of persons with S/PIMD through an online questionnaire (Qualtrics, 2005). To avoid order effects in the results, the designs were presented in a random order to each participant. Participants were asked to assess each design on several criteria on a scale from 0 to 10. Examples of those criteria are: “this is an accurate representation of pain”, “it is clear what this means”, “this is suitable for the user group” and “it is difficult to understand”. Participants were also asked to evaluate whether each design was suitable for people with many distractions, vision impairments, and mono-chromatic vision. All questions are shown in Supplementary Table 1.

As each new design was presented, participants were first asked to freely associate their thoughts on the aspects of the design. If participants responded that they did not think the design visualized pain well, they were asked to elaborate. Results of these open-ended questions were grouped into a word cloud. For the multiple-choice questions either percentages per answer (nominal or ordinal) or averages (interval or scale) were calculated, using Microsoft Excel.

The online questionnaire was completed by 117 participants, with an average age of 45.1 years (SD 11.8, range 21–67) and 95% women. In the Netherlands, 85% of professional caregivers of persons with S/PIMD and approximately 95% of primary caregivers at home are women (CBS, 2019). Professional caregivers had on average 16.2 years of experience (SD 10.3 years, range 0.5–40 years) in the care for persons with severe or profound intellectual disability.

#### 2.5.2 Results

As indicated in Table 4, 71% of respondents found design C a good way to display pain, making this design the best received. Together with Design A, respondents thought this design was intuitively clear, fitting for the target group, not difficult to understand and would prompt them into action more than design B. Design C also was found to best contrast between pain and no pain. Respondents thought design B was unintuitive and difficult to understand, but it created the least (visual) stress. Most participants desired all three designs to be changed. For design C they wanted another shape and movement, but similar colours. Design B needed a simpler shape, more contrast and less movement. For design A respondents recommended a different shape, more movement and other colours.

**Table 4:** Numerical results from the online questionnaire in phase two of the design process.

Design	Good	Clarity		Fitting	Understanding		Action	Stress	Change
		Difficult	Intuitive		Pain	No pain			
A (n = 111)	57%	3.43	5.58	6.05	6.79	5.47	5.71	3.28	74%
B (n = 116	19%	5.93	2.21	2.31	2.67	2.63	2.61	2.61	95%
C (n = 112)	71%	3.76	5.29	5.67	6.72	6.17	5.73	5.73	61%

**Figure 10:**
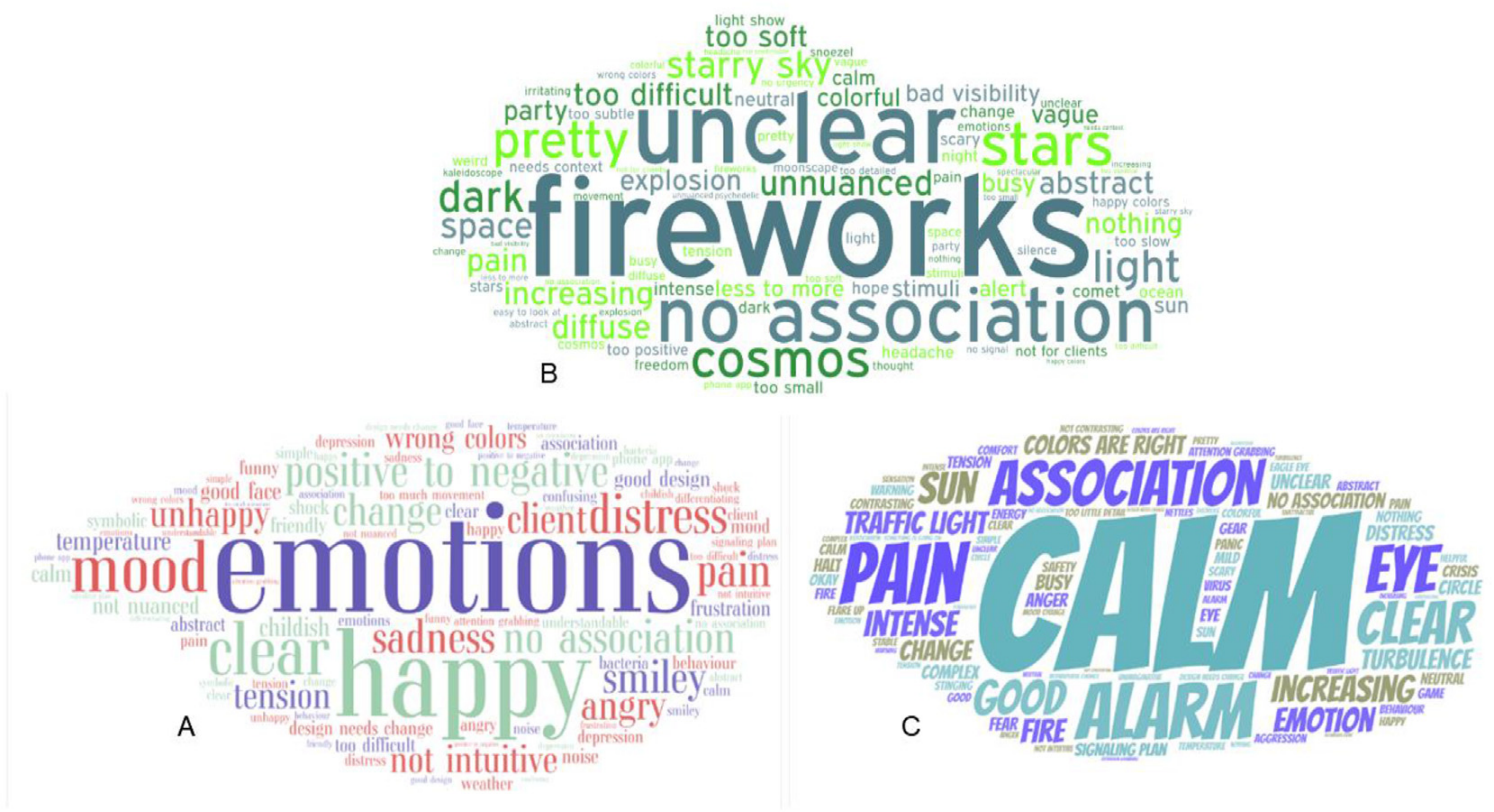
The answers to the open-ended questions from the online questionnaire were translated and transformed into word clouds for each design. The letters on the bottom left of each word cloud represents the design it relates to.

Respondents were also asked for their first thought when seeing the design and why they thought this design was not a good visualization for pain. Responses to these open questions are displayed in Word clouds per design in Figures 10a, b, and c. Results indicate that design A reminded the respondents most of emotions but not pain. They saw happiness and distress, anger, tension, unhappiness, and sadness. The positive to negative change was clear and the design reminded them of their clients, but they mentioned that the design was not intuitive for pain and had incorrect colours. Design B was associated with fireworks, stars, a starry sky, the cosmos, space, an explosion and a light show. Respondents were unclear about what this design should visualize and had no association with pain. They found the design too positive, too easy to look at, too pretty, vague, abstract, too difficult and soft to display something like pain. The respondents thought design C was good and clear. It reminded them of calm and alarm, and they associated this design most with pain. Design C also reminded them of an eye, the sun, traffic light, and fire, though the colours were good. It displayed intensity and turbulence, and clearly visualized an increasing change.

### 2.6 Phase three: discussion of phase two results with an expert focus group to create a final design that encompasses all important aspects for the user group

#### 2.6.1 Methods

Via videoconference, results from the online questionnaire from phase two were discussed by another focus group of six experts. All members of this group were aware of the design's function and the end-user. The goal of this focus group was to decide, based on questionnaire results, which design was most adequate and whether and how this design could be adjusted.

The focus group was organized as follows: One of the three designs was shown, via screen sharing. Participants were invited to say anything that came to mind when viewing the design. Hereafter the results from the questionnaire were presented, starting with the results from multiple choice and numeric questions (e.g. ‘This design is the most difficult to understand compared to the other two designs’ or ‘Participants think that this design shows the best display of pain’). Then common statements given in the questionnaire were shared and focus group participants were asked whether they agreed with these participants. Hereafter, the focus group was asked to discuss the design changes offered by questionnaire participants. And, due to the COVID-19 pandemic at that time, focus group participants were specifically asked whether one of the designs made them think of some of the used images of COVID-19, which was a question that was omitted in the online questionnaire since this was distributed before COVID-19 reached pandemic proportions. The three designs were shown to the focus group in a random order, to minimize the influence of the preferred order of the researcher.

At the end of the focus group discussion, the experts were asked which design they preferred as the best display of pain and whether this design needed to be adjusted, based on statements from the questionnaire or their own opinion. Focus group participants could either say they thought the design was suitable as is, or that adjustments were necessary, based on the discussion. The focus group experts were asked to vote and choices were made based on a two-third-majority agreement. The entire focus group discussion was audio-recorded.

#### 2.6.2 Results

Focus group participants agreed with questionnaire participants that colours used in design A were off-target. Grey was more associated with sadness and yellow and grey contrasted insufficiently: The grey warning signal could attract equal attention as the yellow sun, which is undesirable. The focus group found using emoticons very intuitive and easy to understand, though the negative face was associated more with sadness than pain. A grimacing face with contracting eyes and mouth was recommended, which derived from literature on pain observations (Van der Putten and Vlaskamp, 2011) and the revised Faces Pain Scale (Hicks et al., 2001).

The focus group agreed with the questionnaire participants on design B being unintuitive and difficult to understand. They found the design beautiful, and too ‘easy’ to look at. This design would not cause stress in the end-user but a small amount of stress was deemed necessary to prompt caregivers into quick pain relief action.

The expert group agreed with the questionnaire participants that design C intuitively visualized pain better than the other designs. The colours and shape changes created a good contrast, and the negative side would attract more attention than the positive side. Possible stress the design might cause did not worry them, though they agreed that the negative side had a resemblance to the visualization of the COVID-19 virus.

**Figure 11:**
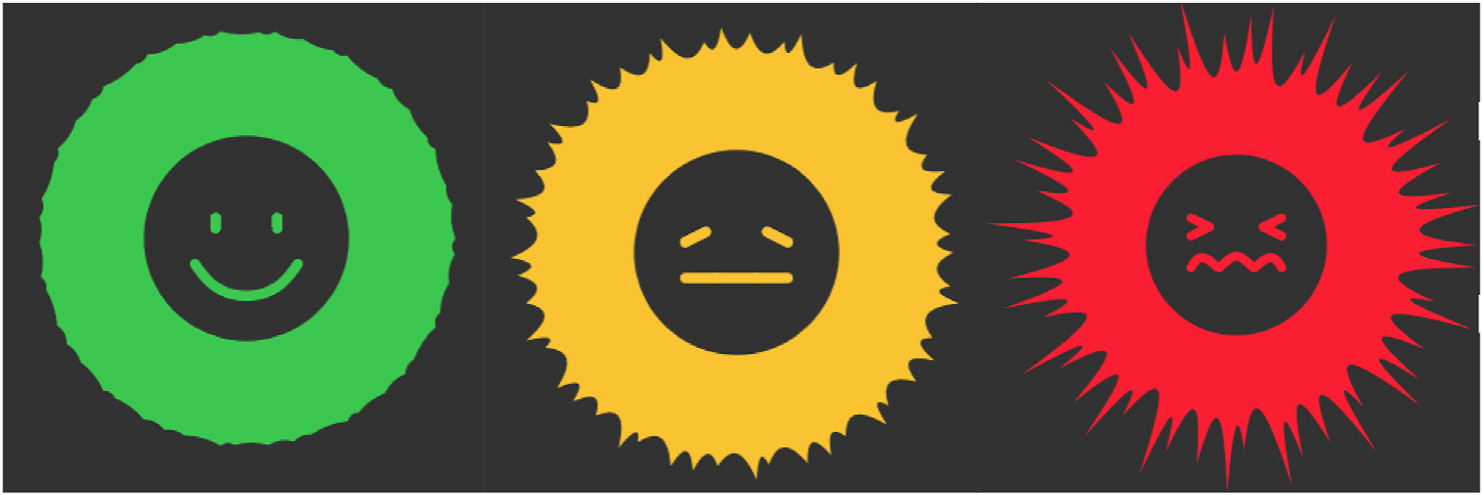
This final design transforms dynamically from a green, happy face, via an orange, neutral face, to a red, painful face. All designs presented in this paper can be viewed here.

After discussing all designs, expert group participants voted on two queries; which design should be continued and should it then be changed? There was a unanimous agreement to continue with design C and another unanimous agreement to change this design to increase clarity and lessen the resemblance to a virus. An addition of a face inside the ring was furthermore unanimously agreed upon, using a smiling and grimacing face (Figure 11).

## 3 Discussion

A co-creative design process is described for a visualization of pain of clients who cannot self-report, to be used in daily caregiving. Three designs were created, evaluated in three phases, and finally adjusted to reach a design that encompasses the wishes and demands of the end-users. The process shown is particularly suited for emotion-related design tasks with the two simultaneous difficulties that the key stakeholder is unable to provide input and that the validation during later use is expected to be tedious. The goal of the process was to make ambiguous data on pain unambiguous and easy to understand. Under such circumstances, it is essential that as much available knowledge and experience from various sources is utilized. And of course, the existing knowledge on pain, notably from literature, should be used. Several design methods and user groups were combined to generate a creative as well as a systematic multi-disciplinary design process.

The subjective experience of pain resonates with other areas of design theory and practice, where both the subjective first-person perspective and the second and third-person perspectives are needed. We argue, that creating visualizations or tangible experiences with such an elusive and ill-defined experiences as pain and consciousness (Price et al., 2002) might benefit from the visualization of both as proposed by Frederiks et al., (2015) as a tool that can facilitate understanding of the interplay between the different perspectives in designs that should be used by several stakeholders. The visualization for pain in the system attempts to represent the first-person perspective of the patient, and should encompass all requirements to make it useful in daily care. The three-step design process described in this article aimed to address several design challenges and resulted in a visualization encompassing all requirements from the user groups. The process also utilized a multi-disciplinary approach, giving end-users, both parents and professional caregivers, the tools to provide invaluable input at each step.

All three phases of the design process provided results on thoughts, feelings, and needs of professional caregivers and parents and the challenges they face in the daily care of a person or persons unable to communicate about their own experience of pain. With respect to associations to pain, we found that this niche group of end-users did not differ much from pain patients in their associations, though the resulting z-scores did indicate that pain associations that were possibly deemed obvious by many, such as the colour red, sharp edges and hardness, were not unanimously selected to represent pain by this group. Regarding workplace necessities, it was discovered that in an environment with many sounds, movements and distractions, a visualization should have the potential to attract the attention of the caregiver when necessary and stay neutral when no pain is experienced. Results from the literature search indicated that a warm/cool contrast regarding colours and a sharp/rounded contrast regarding shape would be quite useful to indicate the continuity from pain to no pain.

The designs created in the first phase of the process, shown in Figures 5, 8, and 9, were then further evaluated by more than a hundred parents and professional caregivers in an online questionnaire. Among many unique opinions on the usability of these three designs, many commonalities were also found. End-users were most positive about the usefulness of design C (Figure 9) as a representation of a pain experience and found design B (Figure 8) to generate the least amount of stress in the end-user. The face used in design A (Figure 5) was deemed the most intuitive, though the suggestion was made that the negative side represented sadness more than pain. With these evaluations and opinions, partially displayed in the word clouds of Figure 10, it was shown that in the three design processes from the first phase unique information was gathered, which was then evaluated in a 90-min focus group discussion that resulted in a final design (Figure 11), that combined an adaptation of the intuitive face of design A and the colours and shape of design C, creating something that would attract attention and motivate caregivers to react quickly and was simple to comprehend.

The design process shows how valuable the collaboration of end-users can be, especially when these end-users are a niche group on which very few studies focus. The authors have found no earlier studies that looked at pain associations within this user group. The resulting design created in this study will be integrated in a mobile application to be used by caregivers and parents of those with severe or profound intellectual and multiple disabilities. Next steps will be to create this app, test it in the target group and evaluate its usability with the end-users.

### 3.1 Limitations and strengths

While care was taken to conduct an extensive and thorough design study, there are limitations. In the first phase of this design study, small groups were assembled to collaborate with the researchers. Also, many of the participants were recruited from the networks of the authors, which may limit the variation of their opinions. However, all participants were very involved in the study and determined to co-create something useful for daily caregiving, providing quality input on all aspects. Also, thanks to the online nature of some aspects of the study, participants hailed from all corners of the country and had varied expertise (see Table 1). As a result, the participation in the first phase led to three designs encompassing desires, demands and needs of end-users, confirmed by a large number of participants in the second phase.

While semi-structured interviews could have provided more comprehensive information and design evaluations, we doubt whether this would have given a better representation of the end-users than the eventual participant group of phase two. The six expert focus group of phase three of the process thoroughly ran through and endorsed results from the online questionnaire and came to a unanimous agreement. Of course, further tests in daily practice should show whether this design fully subscribes to the wishes and demands of caregivers.

In this design study a variety of design aspects was used, ranging from semantic differential comparisons to a brainstorming session, using both end-users and design experts, to join co-creative development with proven design methods. The end-users formed a varied group with various levels of domain knowledge and design experience, though undoubtedly included those that had trouble with the semantic differential method or who did not fully grasp for whom they were making decisions (themselves or their clients). Furthermore, the design experts involved in the brainstorm session might not fully have been able to imagine the needs of the end-user, but the way in which several design methods were combined while involving the end-user in several different manners should minimize the effect of this limitation.

## 4 Practical implications

This study describes a process for designing visualizations of subjective (first-person) experiences, such as the experience of pain, which may have far-reaching consequences for design practice. The phenomenological approach in design, which takes a first-person perspective contrasts with empirical science and clinical observations, often referred to as a third-person perspective (Thacker & Moseley, 2012). Here we see a new field for the design theory and practice to help to bridge the gap between the two perspectives by visualizing or materializing these perspectives and converging them in a solution that in our case can improve the understanding of the well-being of a client unable to communicate and ease the emotional workload of a parent or professional caregiver. In the current study end-users were extensively involved, in many different roles, which gave them the opportunity to evaluate each design phase but also enlarged their involvement in the eventual product. In its core, the design task is the mapping from pain levels, which are emotional and subjective in nature, to visualizations which should convey the right semantics and intuitive associations. This core task is not solvable by pure logic and functional reasoning alone. This means that the sensitivity and creativity of trained designers should be used, while it is essential to give a voice to the caregivers and family, being both stakeholders and expert-proxies to the people with severe or profound intellectual and communicative disabilities.

## 5 Conclusion

The described process was designed in an eclectic manner, choosing state-of-the-art sub-methods to optimally use resources and optimize quality by working in small steps, not losing valuable options, and by careful decision making, balancing functional and emotional aspects. Lessons from the study and results from its systematic process could be extended to design for other non-verbal groups, such as neonates and comatose patients, for which similar challenges exist. One could also think of involving end-users in design for bio-feedback, which is currently used in graphs and games, but could be extended to health and care.

## Declarations

### Author contribution statement

Helen Korving: Conceived and designed the experiments; Performed the experiments; Analyzed and interpreted the data; Contributed reagents, materials, analysis tools or data; Wrote the paper.

Paula Sterkenburg, Professor: Conceived and designed the experiments; Analyzed and interpreted the data; Contributed reagents, materials, analysis tools or data; Wrote the paper.

Emilia Barakova, Doctor; Loe Feijs, Professor: Conceived and designed the experiments; Contributed reagents, materials, analysis tools or data; Wrote the paper.

### Funding statement

Paula Sterkenburg was supported by 10.13039/501100001826ZonMw [8084 5009 8345].

### Data availability statement

Data will be made available on request.

### Declaration of interest's statement

The authors declare no conflict of interest.

### Additional information

No additional information is available for this paper.

## Appendix A Supplementary data

Pain visualisation_Supp Table 1_V2.docx Pain visualisation_Supp Table 1_V2.docx
