# Supplementary material for: Designing pain visualisation for caregivers of people with special needs: A co-creation approach
Source: Heliyon. 2022 Dec 5;8(12):e11975. doi: 10.1016/j.heliyon.2022.e11975 (PMC9730134; doi:10.1016/j.heliyon.2022.e11975)
Supplement: Pain visualisation_Supp Table 1_V2.docx [file mmc1.docx]

Supplementary Table 1. Questions from the online questionnaire used in phase 2 of the study.

| # | Question | Answer options |
| --- | --- | --- |
| 1 | Informed consent | - I read the information and consent to filling out the questionnaire |
| 2 | In what manner are you involved in the care for persons with S/PIMD? | - As a parent - As another family member - As daily caregiver - As behavioural therapist - As physical therapist - As medical expert - As speech therapist - In another manner: |
| 3 | Only for professionals:  How many years have you been involved in the care for persons with S/PIMD? | Fill out number of years: |
| 4 | What is your gender? | - Female - Male - Other - I’d rather not say |
| 5 | What is your age? | Fill out number of years: |
| *One of the designs is shown as a moving gif* | | |
| 6 | What are your thoughts upon seeing this design? | Fill out your answer: |
| 7 | Do you consider this design to visualise no pain to pain in a good way? | - No - Yes |
| 8 | If no: why don’t you think this design visualises no pain to pain in a good way? | Fill out your answer: |
| 9 | On a scale of one to ten:  How well do you think this design fits the following statements | - It is difficult to understand - It is clear what it means - It clearly distinguishes between levels - It fits the target group (those involved in the care for persons with S/PIMD) - It gives me an uncomfortable feeling - If the design is at the pain level, it is clear to me that is represents ‘pain’ - If the design is at the no pain level, it is clear to me that it represents ‘no pain’ - It urges me into action |
| 10 | Do you consider this design suitable to be used by the following: | - Those that have a vision impairment - Those that have trouble or an inability to distinguish colours - Those that work in a highly distracting environment - None of the above |
| 11 | If you could change anything in this design, what would that be? | - Different shape - More movement - Less movement - Different colours - No colours (black and white) - No colours (grey) - Simpler shape - No change is necessary |
| *Another design is shown as a moving gif and the questions repeat from #6* | | |
